# Supplementary material for: Derailing the aspartate pathway of Mycobacterium tuberculosis to eradicate persistent infection
Source: Nat Commun. 2019 Sep 16;10:4215. doi: 10.1038/s41467-019-12224-3 (PMC6746716; doi:10.1038/s41467-019-12224-3)
Supplement: Supplementary file 1 — Supplementary info [file 41467_2019_12224_MOESM1_ESM.pdf]

Supplementary information for:

Derailing the aspartate pathway of *M. tuberculosis* to eradicate persistent infection

Hasenoehrl *et al*.

Supplementary Information

Strains and Plasmids	Description	Reference
	Strains	
M. tuberculosis H37Rv	*Mycobacterium tuberculosis* H37Rv	
Mtb Δ*metX*	H37Rv in which *metX* (formerly *metA*) was deleted by specialized transduction and the hyg^r cassette was unmarked	Berney et al. 2015 [1]
Mtb Δ*thrA*	H37Rv in which *thrA* was deleted by specialized transduction and replaced with a hyg^r cassette	This work
Mtb Δ*thrB*	H37Rv in which *thrB* was deleted by specialized transduction and replaced with a hyg^r cassette	This work
Mtb Δ*lysE*	H37Rv in which *lysE* was deleted by specialized transduction and replaced with a hyg^r cassette	This work
Mtb Δ*lysG*	H37Rv in which *lysG* was deleted by specialized transduction and replaced with a hyg^r cassette	This work
Mtb Δ*metX*::DUC1	Kan^r $Strep^r$ derivative of Δ*metX* in which atc induces repression of *metX* by T38 and degradation of MetX by SspB (Dual Control) with the native shine delgarno (Supplementary Table 2)	This work
Mtb Δ*metX*::DUC5	Kan^r $Strep^r$ Hyg^r derivative of Δ*metX* in which atc induces repression of *metX* by T38 and degradation of MetX by SspB (Dual Control) with the SD5 shine delgarno (Supplementary Table 2)	This work
Mtb Δ*thrA*::DUC3	Kan^r $Strep^r$ Hyg^r derivative of Δ*thrA* in which atc induces repression of *thrA* by T38 and degradation of ThrA by SspB (Dual Control) with the SD3 shine delgarno (Supplementary Table 2)	This work
Mtb Δ*thrA*::DUC5	Kan^r $Strep^r$ Hyg^r derivative of Δ*thrA* in which atc induces repression of *thrA* by T38 and degradation of ThrA by SspB	This work

	(Dual Control) with the SD5 shine delgarno (Supplementary Table 2)	
Mtb Δ*lysE*::comp	Kan^r derivative of Δ*lysE* complemented with pMV361-LysE which expresses *lysE* under the hsp60 promoter	This work
	Plasmids	
pGMCgS-TSC10M1-sspB	Strep^r; contains PtetO-*sspB* and tsc10; integrates into att-giles	Schnappinger et al. 2015 [2]
pGMCKq19-T38S38-P750-metX-DAS+4	Kan^r; contains qtag19, PtetO-4C5G-metX-DAS+4, and tetR38; integrates into att-L5.	This work
pGMCKq19-T38S38-P750-thrA-DAS+4	Kan^r; contains qtag19, PtetO-4C5G-*thrA*-DAS+4, and tetR38; integrates into att-L5.	This work
pMV361-LysE	*AttL5* integrative *E. coli*-*Mycobacteria* shuttle vector harboring *lysE* under expression of an hsp60 promoter	This work

Supplementary table 1. Bacterial strains and plasmids

Specialized Transduction Primers	
name	sequence
thrB_LL	TTTTTTTTCCATAAATTGGGAGGCACAGCAGCAGTCCAA
thrB_LR	TTTTTTTTCCATTTCTTGGTCGTGAACTTTAATTCGCGGGATGTCTCACTGAGGTCTCTACCAGCCCAGAAGGCAACAA
thrB_RL	TTTTTTTTCCATAGATTGGAGTAGAATCGGTGACTGACACGAGTGTCTGGTCTCGTAGCGCAAAGGGATTTGCCGTTA
thrB_RR	TTTTTTTTCCATCTTTTGGGTCGCCCTTGTCGTGCTCTT
thrA_LL	TTTTTTTTCCATAAATTGGCCCGAAAAGACGGCACAGAT
thrA_LR	TTTTTTTTCCATTTCTTGGTAGAATTATTGCACAGGCGGGATGTCTCACTGAGGTCTCTTCTCGATGATGCGGACAACC
thrA_RL	TTTTTTTTCCATAGATTGGAGATGTAGCAAGGTCTGACACGAGTGTCTGGTCTCGTAGACCGTTGATGCACTGGACGA
thrA_RR	TTTTTTTTCCATCTTTTGGCTCGAACGCTGCCGTTTTCT
asd_LL	TTTTTTTTCCATAAATTGGCCATGGAAGACCCCATCCTG
asd_LR	TTTTTTTTCCATTTCTTGGGATGTGATTAACGTCGTCGTGATGTCTCACTGAGGTCTCTCAACGTGCGCATGACCTGAC
asd_RL	TTTTTTTTCCATAGATTGGAGGAGTCTCATACGTGATAGCGAGTGTCTGGTCTCGTAGTCGTGTCGGGGGATAACCTG
asd_RR	TTTTTTTTCCATCTTTTGGGCAGGTCCTTGGTGGTGGTC
lysE_LL	TTTTTTTTCACAAAGTGCCTGCTGGCCACTGGTAGGT
lysE_LR	TTTTTTTTCACTTCGTGCCATGTATCAGATCCTGGGTGATGTCTCACTGAGGTCTCTGGCGATCAGCGTGAAGCAG
lysE_RL	TTTTTTTTCCATAGATTGGACTCTGAATCGAAGTTTGCCCGAGTGTCTGGTCTCGTAGGAGAATCCTCGACGGCCTGA
lysE_RR	TTTTTTTTCCATCTTTTGGCGAGTCCGAGGATGGCAACT
lysG_LL	TTTTTTTTCCATAAATTGGGTGGCGAACCATACCGCACT
lysG_LR	TTTTTTTTCCATTTCTTGGGCTTGATGGAATGTCTCCTGGATGTCTCACTGAGGTCTCTAAGCTGCCCAGTTCGACCAC
lysG_RL	TTTTTTTTCCATAGATTGGGGACAGCATGTGTGAAATCCCGAGTGTCTGGTCTCGTAGGCGAATTACCGACACGGTGAG
lysG_RR	TTTTTTTTCCATCTTTTGGCGAGGTAAGCGAGTCGACGAA
**Confirmation Primers**	
name	sequence
Uni Uptag	AGAGACCTCAGTGAGACATC
thrB_L	GAGGCACAGCAGCAGTCCAA
thrB_R	ACCAGCCCAGAAGGCAACAA
thrA_L	CCCGAAAAGACGGCACAGAT
thrA_R	TCTCGATGATGCGGACAACC
asd_L	CCATGGAAGACCCCATCCTG
asd_R	CAACGTGCGCATGACCTGAC
lysE_L	CCTGCTGGCCACTGGTAGGT
lysE_R	CCTGCTGGCCACTGGTAGGT
lysG_L	GTGGCGAACCATACCGCACT
lysG_R	AAGCTGCCCAGTTCGACCAC

Conditional Knockdown Constructs Primers	
name	sequence
MetA_FWD_P1	GGGGACAGCTTTCTTGTACAAAGTGGAGGAATTCTGACATGACGATCTCC
MetA_REV_P2	TCTCGGAGTAGTTCTCGTCGTTGGCGGCCTTATCGTCATCGTCCTTGTAGTCCCGCCGACACGCG
P3	GGGGACAACTTTGTATAATAAAGTTGCTAGCTGGCGTCCGCGTAGTTCTCGGAGTAGTTCTCGTCG
Asd_P1_FWD	GGGGACAGCTTTCTTGTACAAAGTGGACGGTGTACGCGGGG
Asd_P2_REV	TCTCGGAGTAGTTCTCGTCGTTGGCGGCCTTATCGTCATCGTCCTTGTAGTCCAAGTCGGCGGTCAGC
ThrA_P1_FWD	GGGGACAGCTTTCTTGTACAAAGTGGAGTTTGGAAGTGAGGTGACCC
ThrA_P2_Rev	TCTCGGAGTAGTTCTCGTCGTTGGCGGCCTTATCGTCATCGTCCTTGTAGTCTAAGCCGGTTCCTTCCAGTCG
pGMCK-MetA-SD2-F	[Phos]ACGAATTCTGACATGACGATCTCCG
pGMCK-MetA-SD3-F	[Phos]AGCAATTCTGACATGACGATCTCCG
pGMCK-MetA-SD4-F	[Phos]AGGCATTCTGACATGACGATCTCC
pGMCK-MetA-SD5-F	[Phos]CTCTCTTCTGACATGACGATCTCCG
pGMCK-ThrA-SD2-F	[Phos]AGTTTGGAACTGAGGTGACCCG
pGMCK-ThrA-SD3-F	[Phos]AGTTTGGAAGTCAGGTGACCCG
pGMCK-ThrA-SD4-F	[Phos]AGTTTGGAAGTGACGTGACCCG
pGMCK-ThrA-SD5-F	[Phos]AGTTTGGACTCTCCGTGACCCGTG
pGMCK-SD-SDM-Rev	[Phos]CCACTTTGTACAAGAAAGCTGGG

Supplementary table 2: primer list

Δ*metA* Strains	Shine-Delgarno	Δ*thrA* Strains	Shine-Delgarno
SD1 (Native)	AGGAA	SD1 (Native)	AGTGAGG
SD2	ACGAA	SD2	ACTGAGG
SD3	AGCAA	SD3	AGTCAGG
SD4	AGGCA	SD4	AGTGACG
SD5	CTCTC	SD5	CTCTCCG

Supplementary table 3. Shine-Dalgarno sequences of SD scanning mutants

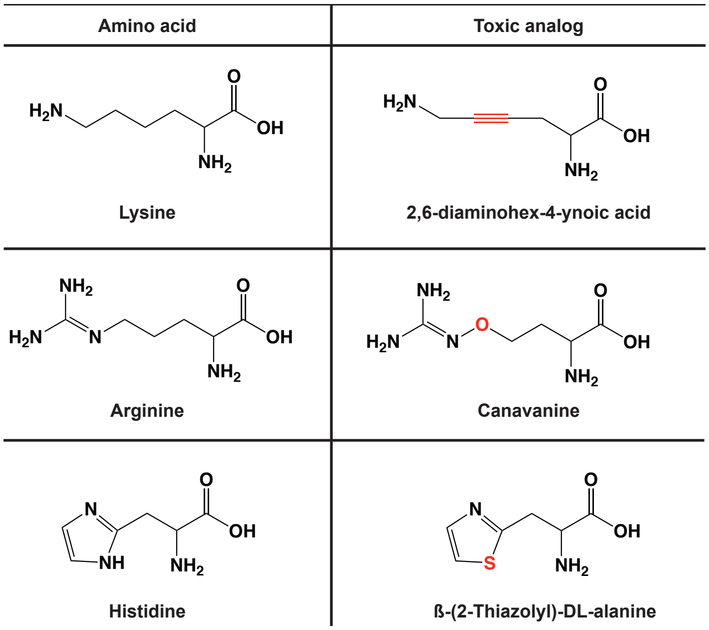

Supplementary Figure 1. Structures of Toxic Amino Acid Analogs

The structures are presented for the amino acids and their toxic analogs.

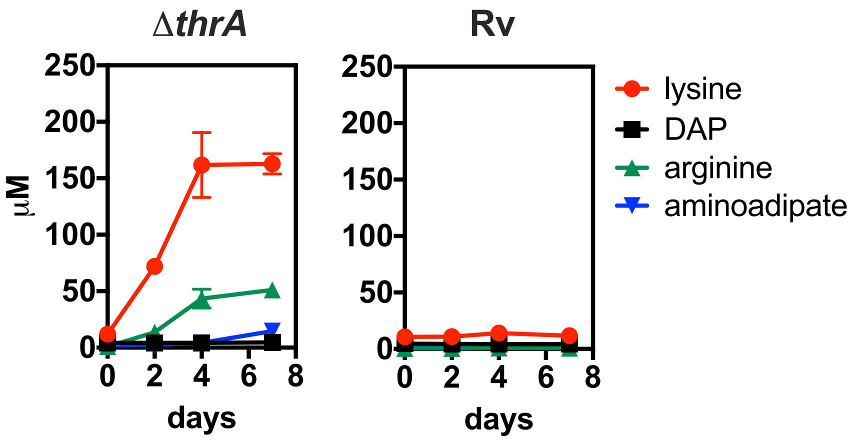

Supplementary Figure 2. Extracellular accumulation of lysine, diaminopimelate (DAP), aminoadipate and arginine. Error bars show standard deviations from n=3 independent biological replicates.

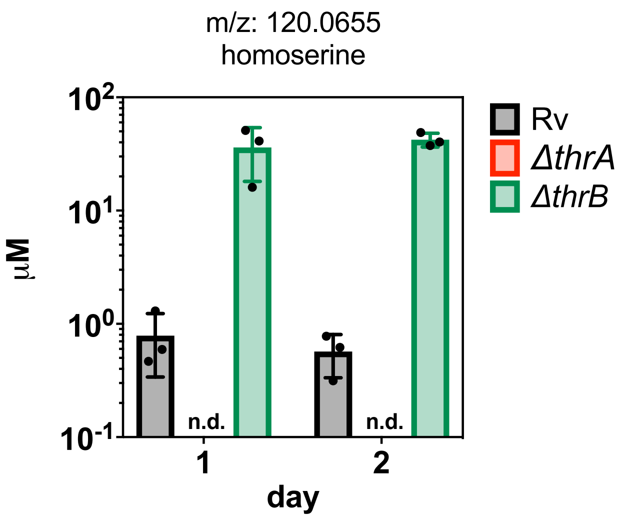

Supplementary Figure 3: Homoserine concentrations in *Mtb ΔthrB* (green bars) are about 50-fold higher than in WT (black bars) and not detectable at all in *Mtb ΔthrA* (red bars). All values are the average of three biological replicates (n = 3) ± s.d. (error bars depict standard deviations) and are representative of a minimum of two independent experiments. ** p-value <0.01 in student t-test. Bars are overlaid with dot plot of individual values.

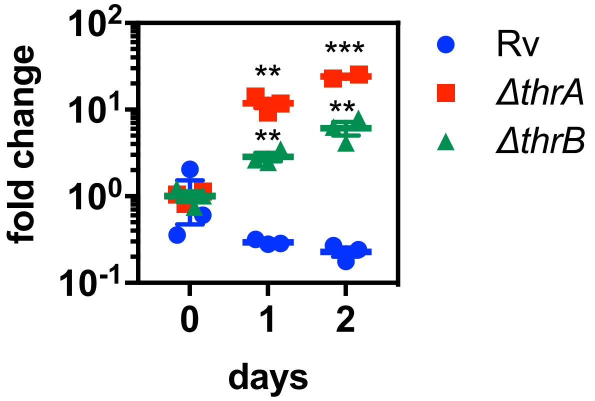

Supplementary Figure 4. Comparison of extracellular lysine accumulation of Rv (blue dots), Δ*thrA* (red squares) and Δ*thrB* (green triangles) strains. Fold changes were calculated based on area under the curve of raw counts normalized to t_0. Results are representative of 3 biological replicates of at least 2 independent experiments. ** p-value <0.01, *** p-value <0.001 in student t-test. Error bars depict standard deviation of n=3 biological replicates.

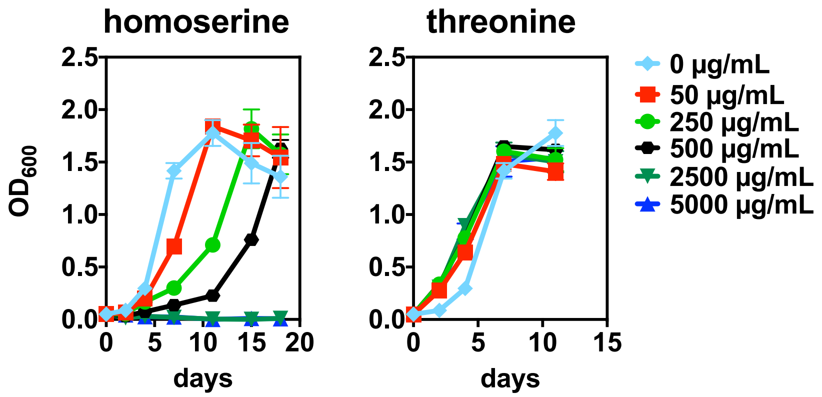

Supplementary Figure 5: Impact of homoserine and threonine on growth of *M. bovis* BCG. Error bars show standard deviations from n=3 independent biological replicates.

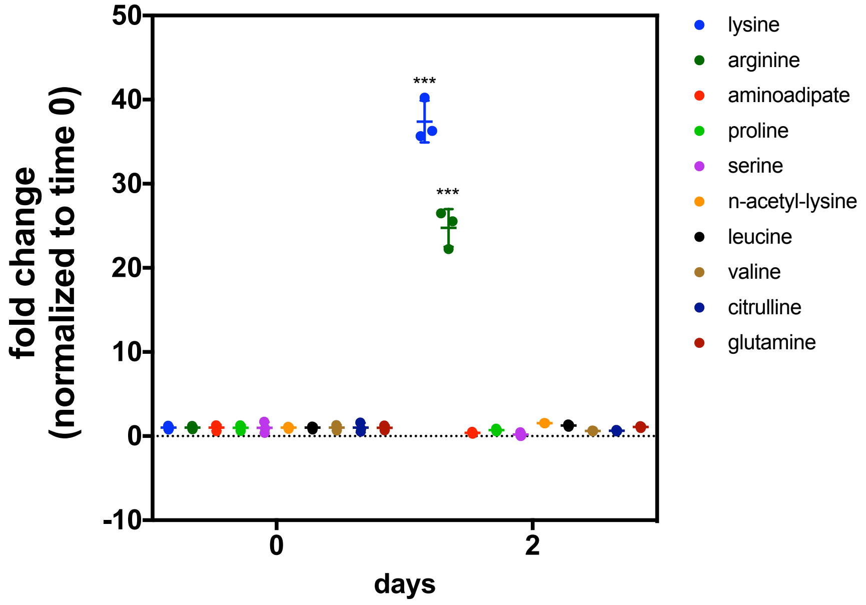

Supplementary Figure 6. Only lysine and arginine abundance changes in the supernatant of *Mtb* Δ*thrA,* indicating that the export of these amino acids is specific and not due to general export/leakage of intracellular metabolites. Results are representative of 3 biological replicates of at least 2 independent experiments. *** p-value <0.001 in student t-test. Error bars represent standard deviation of n=3 biological replicates.

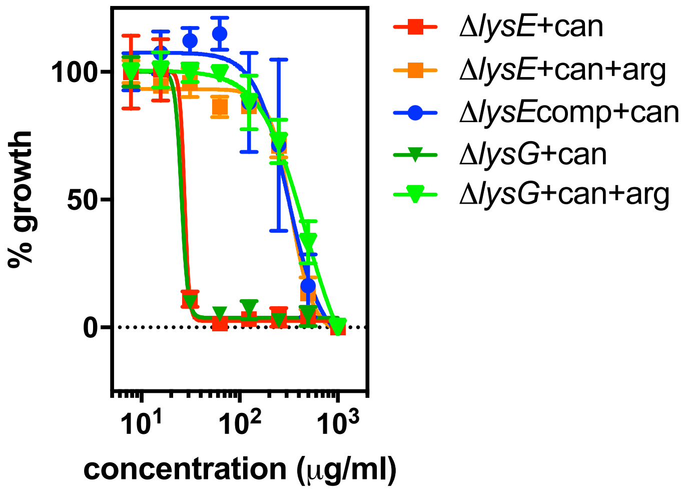

Supplementary Figure 7. Rescue of canavanine toxicity by arginine. MIC of canavanine was determined in strains Δ*lysE*, Δ*lysEcomp*, and Δ*lysG***,** in the presence or absence of 50 *μ*g/ml arginine. Arginine alleviated canavanine toxicity to the level of the Δ*lysE* complemented strain. Error bars show standard deviations from n=3 biological replicates.

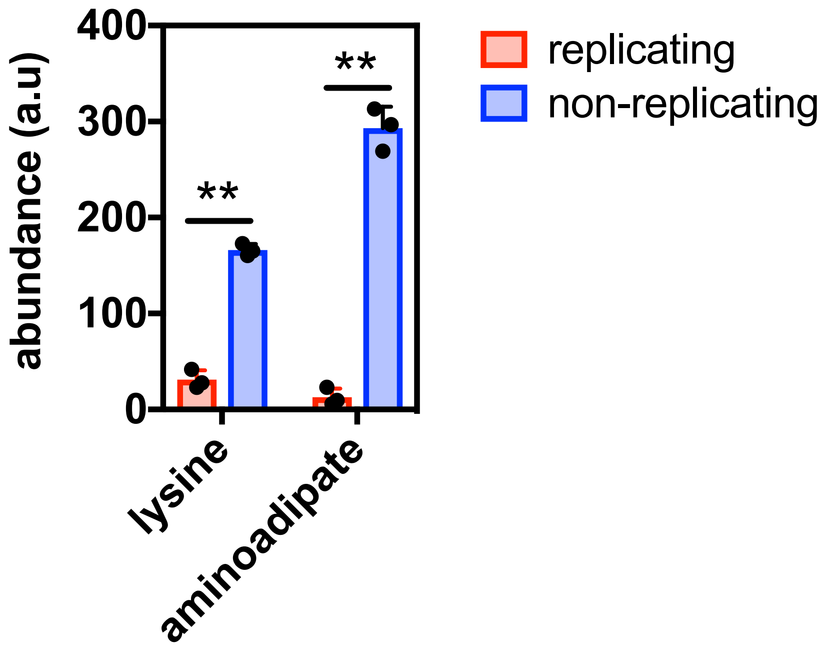

Supplementary Figure 8: Relative abundance of intracellular lysine and aminoadipate in replicating (exponential phase) (red bars) or non-replicating (stationary phase) (blue bars) *M. tuberculosis* H37Rv. Error bars represent standard deviations of n=3 biological replicates (bars are overlayed with dot plot of individual values). ** p-value <0.01 in student t-test.

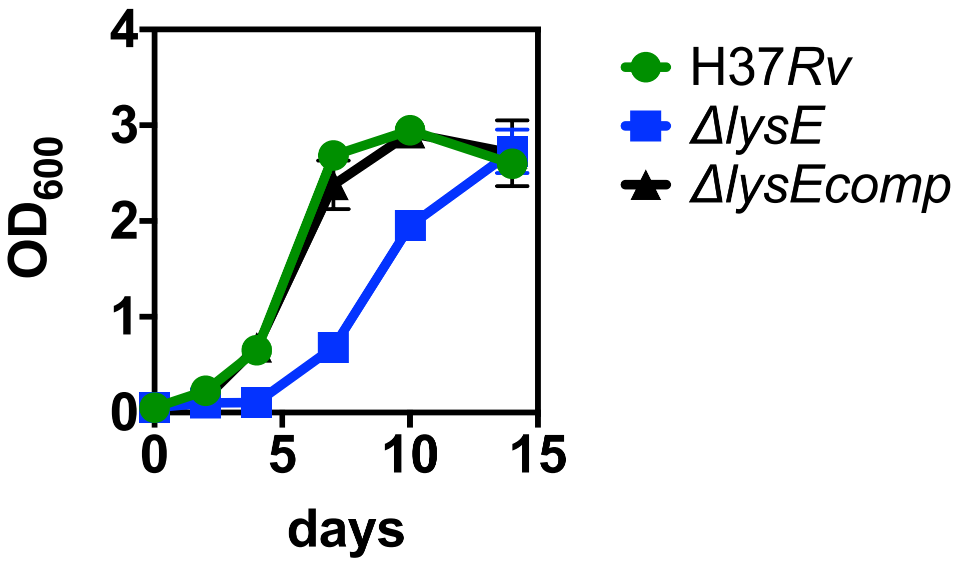

Supplementary Figure 9: Deletion of *lysE* results in delayed exit from lag phase and a reduced growth rate in *M. tuberculosis*. H37Rv (green dots), Δ*lysE* (blue squares), Δ*lysEcomp* (black triangles). All values are the average of three biological replicates (n = 3) ± s.d. and are representative of a minimum of two independent experiments. ** p-value <0.01 in student t-test. Error bars represent standard deviations.

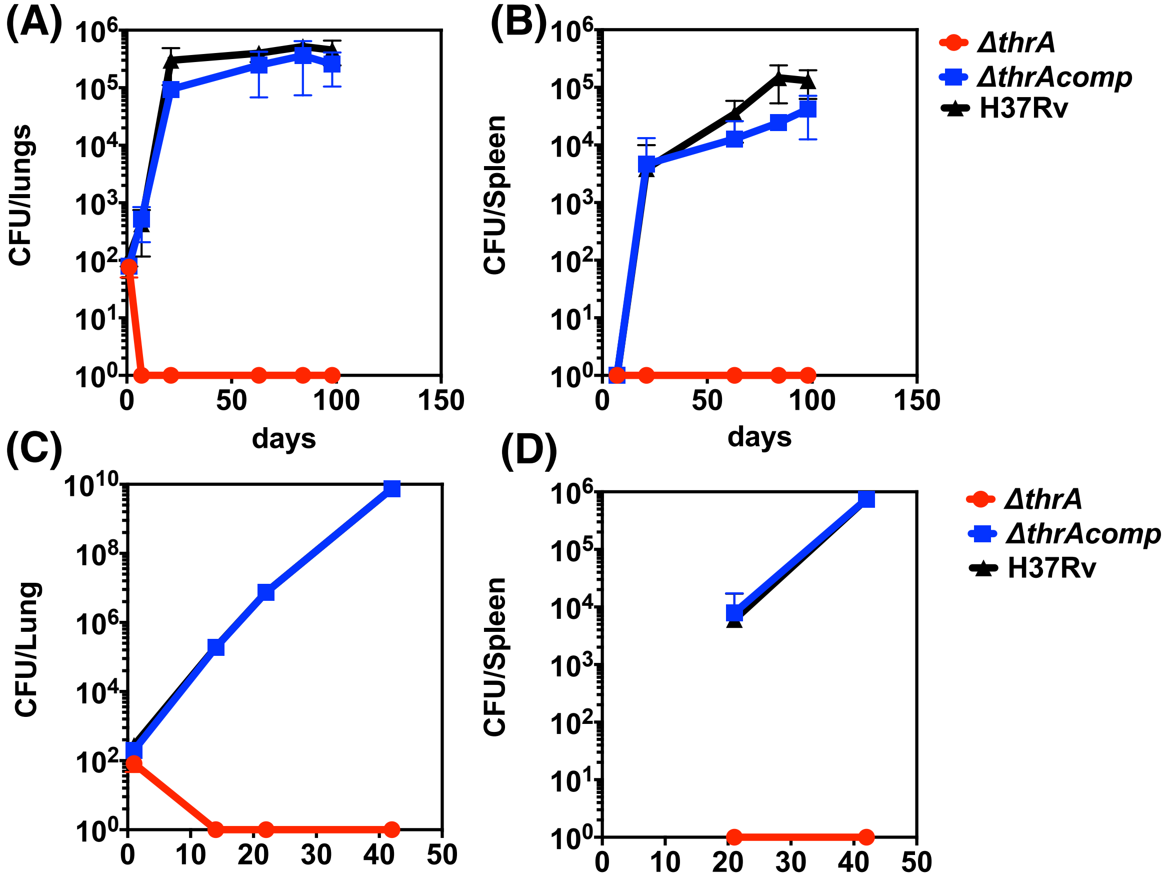

Supplementary Figure 10. *Mtb* Δ*thrA* low dose aerosol infection of C57BL/6 and SCID Mice. C57BL/6 (A,B) and SCID (C,D) mice were infected with Δ*thrA* (red dots), Δ*thrAcomp* (blue squares), and H37Rv (black triangles) with ~100 bacilli per lung. Δ*thrA* was incapable of establishing an infection in either the lungs or spleens of immunocompetent (A,B, respectively) or immunocrompromised (C,D, respectively) mice. Error bars show standard deviations from n=4 mice.

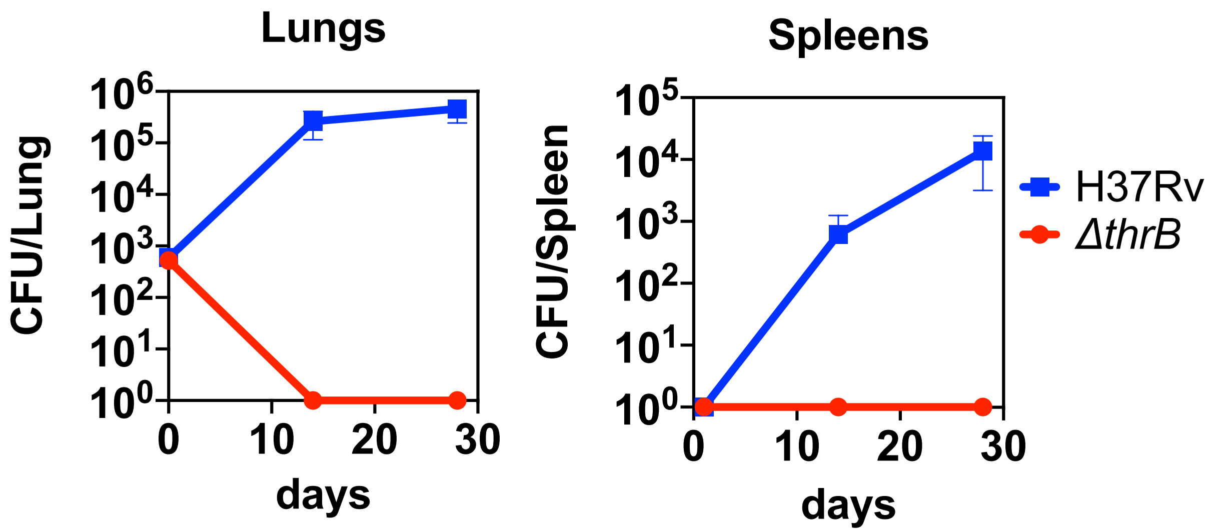

Supplementary Figure 11. *Mtb ΔthrB* aerosol Infection of C57BL/6 Mice

C57BL/6 mice were infected with Δ*thrB* (red dots), H37Rv (blue squares) with ~800 bacilli per lung. *Mtb* Δ*thrB* was incapable of establishing an infection in either the lungs or spleens of C57BL/6 mice. Error bars show standard deviations from n=4 mice.

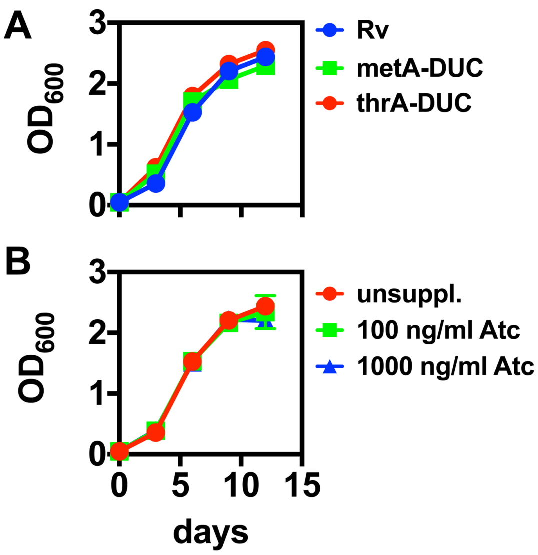

Supplementary Figure 12: Growth curves of *Mtb* H37Rv and DUC strains in absence and presence of anhydrotetracycline (atc). (A) Growth of DUC strains in the absence of atc is indistinguishable from wild-type. Error bars represent standard deviations of three independent biological replicates. (B) *Mtb* H37Rv growth is not affected by up to 1000 ng/ml atc. Error bars show standard deviations from n=3 biological replicates.

Supplementary References

1. Berney, M., *et al.* Essential roles of methionine and S-adenosylmethionine in the autarkic lifestyle of Mycobacterium tuberculosis. *Proc Natl Acad Sci U S A* **112**, 10008-10013 (2015).
2. Schnappinger, D., O'Brien, K.M. & Ehrt, S. Construction of conditional knockdown mutants in mycobacteria. *Methods Mol Biol* **1285**, 151-175 (2015).
