# Supplementary material for: Derailing the aspartate pathway of Mycobacterium tuberculosis to eradicate persistent infection
Source: Nat Commun. 2019 Sep 16;10:4215. doi: 10.1038/s41467-019-12224-3 (PMC6746716; doi:10.1038/s41467-019-12224-3)
Supplement: Supplementary file 3 — Description of Additional Supplementary Files [file 41467_2019_12224_MOESM3_ESM.docx]

**Description of Supplementary Files**

**File Name: Supplementary Data 1**

**Description:** Raw data from metabolomics experiments and metabolite standards. Abundance alues are area under the curve. Standards values include retention time of standards alone and in samples, theoretical expected mass and the actual mass as measured on the mass spec.

**File Name: Supplementary Data 2**

**Description:** Comparison of ∆thrA, ∆thrB, and ∆metX transcriptional responses to auxotrophic starvation. Comparison of the transcriptomic response at day 2 of the ΔthrA and ∆thrB and ∆metX14 mutants during homoserine, threonine, and methionine starvation, respectively. Scale is represented in log2-fold change compared to day 0 in supplemented medium. Genes analyzed in Ref. 13 and of the aspartate pathway analyzed in this paper are presented. Conditional formatting for Log2-fold change values is from -4 (blue) to 4 (red), and for P-values is 0.01-0.05 (light green) and <0.05 (dark green). p-values for each gene and timepoint can be accessed via the GEO database (accession no. GSE119105, GSE119106, GSE119107, and GSE67843).
